# In Vitro Antioxidant Potential of Various Plant Extracts and Application of the Extracts With the Highest Antioxidant Activity in Ground Beef for Enhancing the Quality and Shelf Life

**DOI:** 10.1002/fsn3.70709

**Published:** 2025-07-25

**Authors:** Hanife Bayındır, Birol Kılıç, Melis Yıldız

**Affiliations:** ^1^ Faculty of Engineering and Natural Sciences, Department of Food Engineering Suleyman Demirel University Isparta Turkey

**Keywords:** antioxidant, capers, lipid oxidation inhibition, muscle foods, plant extracts, purslane

## Abstract

Plant extracts have gained attention in the meat industry for their potential to improve quality and safety. Limited information is available on the antioxidant potential of purslane (P), arugula (AR), cress (CR), capers (CA), and linseed (L) extracts for application in the meat industry. This study aimed to assess the in vitro antioxidant activity of P, AR, CR, CA, and L extracts, then select those with the highest antioxidant activity (P and CA) and evaluate their influence on lipid oxidation, physico‐chemical, and microbiological features in cooked ground beef. The highest and lowest (*p* < 0.05) ferric reducing antioxidant power (FRAP) were obtained in P (255.70 mM Fe^2+^/g) and L (55.66 mM Fe^2+^/g) respectively, whereas there was no FRAP difference among others. P (18.54 mg GAE/g) and CA (18.52 mg GAE/g) exhibited the highest (*p* < 0.05) total phenolic content. IC_50_ of 1,1‐diphenyl‐2‐picrylhydrazyl (DPPH) scavenging values of P, CA, and AR were 0.02, 0.10, and 0.08 mg/mL, respectively, and lower than those of L and CR (*p* < 0.05). Higher cooking loss was obtained in ground beef with 2% CA or P compared to control (*p* < 0.05). The extract addition generally caused lower pH than control (*p* < 0.05). At the end of storage, both 2% CA and P reduced thiobarbituric acid reactive substances (TBARS) by 55.70% and 37.37%, respectively, and lipid hydroperoxide (LPO) by 58.42% and 51.11%, compared to control. 2% CA caused the lowest TBARS and LPO among all treatments (*p* < 0.05). CA or P generally had no impact on color and textural features. Total mesophilic aerobic bacteria, coliforms, yeast, and mold were not determined in any treatment. The findings indicated that incorporating CA or P extracts into ground beef effectively inhibited lipid oxidation and could be utilized to enhance the quality and shelf life of meat products.

## Graphical abstract

This study aimed to assess in vitro antioxidant activity of P, AR, CR, CA and L extracts, and then select those with the highest antioxidant activity (P and CA) and evaluate their influence on lipid oxidation, physico‐chemical and microbiological features in cooked ground beef. The findings indicated that CA or P extract incorporation in the ground beef exhibited capability for lipid oxidation inhibition and thus can be utilized to enhance quality and shelf life of the meat products.

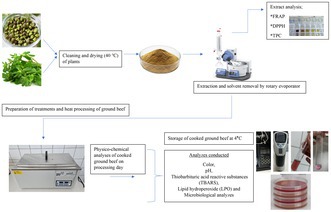

## 1 Introduction

The meat industry has been searching to develop safe, convenient, affordable, enjoyable, and healthy meat products to satisfy consumer demands (Kaynakcı and Kılıç 2009). At the same time, it is important to maintain quality features such as taste and color, and prolong the shelf life (Cmlp et al. 2021). Lipids are the main compounds responsible for the development of many desirable properties of muscle foods (Purriños et al. 2011). Lipids contribute to the taste, texture, and juiciness of the muscle foods (Amaral et al. 2018). Lipid oxidation is one of the vital reactions behind the non‐microbial degradation of muscle foods. Lipid oxidation is the main factor that reduces the consumer acceptability of the meat products due to its negative influences on the quality features by mainly causing deterioration in taste and color characteristics of the product (Hadidi et al. 2022). The primary effects of lipid oxidation on meat products include undesirable changes in odor, taste, color, and texture, as well as reduced protein solubility, water‐holding capacity, and the bioavailability of certain nutrients (Falowo et al. 2014). Lipid oxidation results in the production of free radicals and toxic compounds, such as 4‐hydroxynonenal and malonaldehyde, which are known potential carcinogens and can cause diseases or have adverse effects on consumer health (Csala et al. 2015).

The lipid oxidation is an important issue for the meat industry. The meat industry uses antioxidants to limit lipid oxidation in meat products. Preventing lipid oxidation with antioxidants improves product quality, extends shelf life, and enhances consumer health benefits (Huang and Ahn 2019). Many synthetic antioxidants are costly and have carcinogenic and toxic effects. Due to health concerns, the meat industry favors natural antioxidants over synthetic alternatives (Hadidi et al. 2022). Besides, there is increasing evidence showing a direct link between diet and health, and consumers have embraced this message, resulting in a demand for the meat products produced with natural ingredients (Barden and Decker 2016). To control lipid oxidation in muscle foods, seeds, fruits, bark, leaves, stems, and roots of plants are used as antioxidant sources (Domínguez et al. 2019; Hadidi et al. 2022; Nikmaram et al. 2018). Purees, extracts, or powders of fruits such as grapes, capers, plums, pomegranates, apples, and bananas are also used as antioxidant sources in the meat and meat products (Allaith 2016; Biswas et al. 2015; El Finou et al. 2024; Morsy et al. 2018; Nuñez de Gonzalez et al. 2008). It is known that not only fruits but also vegetables such as potatoes, purslane, arugula, onions, watercress, cabbage, cucumbers, and spinach show antioxidant properties (Amanpour et al. 2024; Chu et al. 2000; Makangali et al. 2024; Shahhoseini et al. 2021). Therefore, natural alternatives such as plant‐based antioxidants are needed to control oxidative reactions in muscle foods (Pateiro et al. 2021).

In the present study, it was first aimed to assess in vitro antioxidant activity of purslane, arugula, cress, capers, and linseed extracts. Furthermore, the second aim of the study was to evaluate efficiency of the extracts (capers and purslane) with the highest in vitro antioxidant activity on lipid oxidation inhibition, physico‐chemical, and microbiological features of cooked ground beef during refrigerated storage.

## 2 Materials and Methods

### 2.1 Materials

Fresh plants (purslane, cress and arugula) and dry plant seeds (capers and linseed) were obtained from the local market in Isparta, Türkiye. 1,1‐diphenyl‐2‐picrylhydrazyl (DPPH), Folin–Ciocalteu's reagent, 2,4,6‐Tri(2‐pyridyl)‐s‐triazine, and 1,1,3,3‐tetraethoxypropane were purchased from Sigma‐Aldrich (St. Louis, MO, USA). Beef *longissimus thoracis et lumborum* muscle, obtained from a local supplier 24 h post‐mortem, was transported to the laboratory in an ice box within 0.5 h to keep the temperature in chilled form.

### 2.2 Preparation of Plant Extracts

Fresh purslane ( *Portulaca oleracea* ), cress (*Lepidium sativum L*.) and arugula (*Eruca vesicaria Mill*.) plants were cleaned from stalks, stems, rocks, and any other foreign objects and washed. After that, the plants were placed in a thin layer on filter paper and dried in an oven (ILD‐EKH‐55, Ildam, Türkiye) set to 40°C for 24 h. During drying, the humidity in the oven was expelled using a ventilation system. Dried plants (purslane, cress and arugula), capers ( *Capparis spinosa* ) and linseed ( *Linum usitatissimum* ) were powdered using a grinder (Arzum, Istanbul, Türkiye). The powders (5 g) were extracted with 50 mL of 80% ethanol (Merck, Darmstadt, Germany) and kept for 24 h at room temperature. Whatman No. 1 filter paper was used to filter the obtained extracts, and a rotary evaporator (Heidolph Instruments Hei‐Vap, Schwabach, Germany) at a speed of 100 rpm and 60°C was used to remove ethanol for 20 min; then the extracts were stored at −80°C (Selani et al. 2011). Additionally, purslane, arugula, cress, capers, and linseed extracts were subjected to ferric reducing antioxidant power (FRAP) evaluations, 1,1‐diphenyl‐2‐picrylhydrazyl (DPPH) radical‐scavenging assays, and total phenolic content (TPC) measurements (Shan et al. 2009).

#### 2.2.1 Total Phenolic Content (TPC) Analysis

TPC analysis was conducted according to the method explained by Papoutsis et al. (2016). Specifically, 1 mL of the tested plant sample was mixed with 5 mL of 10% (vol/vol) Folin–Ciocalteu reagent (Merck, Darmstadt, Germany) and 4 mL of 7.5% (wt/vol) Na_2_CO_3_ (Merck, Darmstadt, Germany). After vortexing and incubation in the dark at 23°C for 1 h, absorbance was measured at 765 nm using a UV spectrophotometer (Varian Australia Pty. Ltd. Victoria, Australia). The obtained TPC results were presented as milligrams of gallic acid equivalents per gram of sample weight (mg GAE/g). A calibration curve for the standard reference was constructed using gallic acid (concentration range from 0.01 to 0.05 mg/mL) as the standard, with the data plotted accordingly.

#### 2.2.2 1,1‐Diphenyl‐2‐Picrylhydrazyl (DPPH) Scavenging Analysis

A 1 mM DPPH radical solution was formed in ethanol. Each test tube received 600 μL of DPPH solution and varying volumes (20–100 μL) of sample extracts. The total volume was adjusted to 6 mL with ethanol; after vortexing, it was incubated in the dark conditions at 23°C for 15 min. Then, the absorbance value measured at 515 nm against a methanol blank using a T8+ UV/VIS Spectrometer (PG Instruments Ltd. Leicestershire, U.K.) was recorded (Dorman et al. 2003).

#### 2.2.3 Ferric Reducing Antioxidant Power (FRAP) Analysis

FRAP assay, based on Soyuçok et al. (2024) involved mixing 300 mM acetate buffer (pH 3.6), 10 mM 2,4,6‐tripyridyl‐s‐triazine (Merck, Darmstadt, Germany) in 40 mmol HCl (Merck, Darmstadt, Germany) and 20 mmol ferric chloride (Merck, Darmstadt, Germany) in a 10:1:1 ratio to prepare the FRAP reagent. Each tube received 100 μL of extract and 3 mL of FRAP reagent; then, each test tube was incubated at 37°C for 30 min. Absorbance was measured against a blank at 593 nm using a T8+ UV/VIS Spectrometer (PG Instruments Ltd. Leicestershire, U.K.). Results were quantified as μmol/L Fe^2+^ based on a standard curve spanning 100–2000 μmol/L Fe^2+^.

### 2.3 Meat Sample Preparation and Design of Experimental Groups

After the addition of 2% NaCl and 10% distilled water, ground meat was kneaded for the duration of 5 min and randomly divided into 5 treatment batches in equal amounts. Each treatment batch per replication contained approximately 1000 g ground meat. The plant extracts with the highest in vitro antioxidant activity (P and CA) were incorporated into the ground beef formulation at 1% and 2% incorporation doses. Five treatments included one control (without any tested plant extract) and four other treatments formed by the incorporation of 1% and 2% P and CA extracts. Then, approximately 50 g ground beef samples were filled in capped plastic centrifuge tubes (50 mL) and cooked in a water bath set at 85°C until the geometric center temperature reached 74°C (Şimşek and Kilic 2024). The core temperature of the samples was systematically controlled using a thermocouple (TK100S, Kimo Instruments, France). After cooking, each cooked ground beef was stored in tubes at refrigerated (4°C) conditions for 10 days. Color, pH, thiobarbituric acid reactive substances (TBARS), lipid hydroperoxide (LPO) and microbiological analyses were implemented during the storage period. Moreover, some physico‐chemical analyses were conducted on the processing day.

#### 2.3.1 Cooking Loss Analysis

The raw ground meat weight for each treatment was recorded before cooking. After cooking, the cooked samples were rolled once on a paper towel to take out the cookout liquid. The cooked ground meat weight was then recorded after cooling to 25°C. The following formula was applied to calculate cooking loss values (Barbanti and Pasquini 2005).

Cooking loss (%) = [(weight of raw ground meat − weight of cooked ground meat)/weight of raw ground meat] × 100.

#### 2.3.2 Physico‐Chemical Measurements

Cooked ground meat samples were analyzed as three replications for proximate composition on processing day. AOAC method (AOAC Method 920.153‐1920 2005; AOAC Method 950.46‐1950 2005; AOAC Method 991.36‐1996 2005) was used to determine the moisture (950.46), fat (991.36) and ash (920.153). Protein (928.08) content was also measured using AOAC method (AOAC 2012). Texture profile analysis (TPA) was performed in triplicate for each treatment at 23°C using a TA.XT2 Texture Analyzer (Stable Micro Systems, UK). Parameters such as hardness (N), adhesiveness (mJ), resilience, cohesiveness, springiness, and chewiness (N) were measured with a probe (aluminum, 5 × 4 cm), using test conditions including a speed of 5 mm/s and compression of 70% with a 50 kg load cell. pH values were monitored daily using a spear electrode (FC 200, Hanna Instruments, Germany) connected to a pH meter (HI 9024, Hanna Instruments, Germany), calibrated with pH 4 and pH 7 buffer solutions. CIE *L* *, *a* *, *b* * values were assessed with a Minolta Colorimeter (CR‐200, D65 Illuminant, Ramsey, USA), calibrated against a white plate, under illuminant D65, 10° observer angle, and SCI mode (Kılıç and Özer 2019).

#### 2.3.3 Thiobarbituric Acid Reactive Substances (TBARS) and Lipid Hydroperoxide (LPO) Analysis

TBARS measurements were conducted two times for each cooked ground beef sample, following the procedures outlined by Özer and Kılıç (2020). The method involves mixing 1 g of cooked ground beef with 6 mL of trichloroacetic acid (TCA) extraction solution containing EDTA and propyl gallate. After homogenization and filtration, 1 mL of filtrate was mixed with 1 mL thiobarbituric acid (TBA) and heated (100°C) for the duration of 40 min. Following centrifugation and cooling, absorbance was read at 532 nm against a blank containing the mixture of TCA (1 mL) and TBA (1 mL). Results were reported as μmol MDA/kg. A standard curve was prepared using tetraethoxypropane.

LPO content was determined as described by Kılıç et al. (2016). Briefly, 1 g of cooked ground beef sample was homogenized in 5 mL of methanol/chloroform (1:1). A 0.5% NaCl solution (3 mL) was incorporated into this mixture and vortexed. After centrifugation (2000 g, 6 min), 1.3 mL ice‐cold chloroform/methanol (1:1) was mixed with the lower phase (the upper phase was carefully removed using a Pasteur pipette), vortexed, and integrated with 25 μL each of ammonium thiocyanate (4.38 M) and iron (II) chloride (18 mM). After 20 min incubation at 23°C, absorbance was read at 500 nm. The results were quantified using a standard curve formed with cumene hydroperoxide (Merck, Darmstadt, Germany), and LPO results were expressed as μmol per kg of meat.

#### 2.3.4 Microbiological Analysis

Cooked ground beef (10 g) was aseptically mixed with the sterile peptone water (90 mL) and homogenized. Peptone water was utilized to prepare serial dilutions. Then, each dilution was plated on specific media: Plate Count Agar (Merck, Germany) for total viable count (TVA) at 30°C for 48 h; Eosin Methylene Blue Agar (Merck, Germany) for coliforms at 37°C for 48 h; and Potato Dextrose Agar (Merck, Germany) for yeast and molds at 25°C for 72 h. Colony counts were carried out after the incubation periods mentioned above (Uşan et al. 2022).

### 2.4 Statistical Analysis

The whole study was conducted with three replications, and the analyses carried out in each replication were also performed in triplicate. The data obtained for pH, CIE *L* * *a* * *b* * color, TBARS, LPO, and microbiological analysis were analyzed by generalized linear mixed model procedure of the Minitab 19.2.0 (Minitab Statistical Software, USA) to determine significant differences during processing and storage stages. In the statistical design, treatments and storage intervals were assigned as fixed effects and replications as a random effect. Significant differences among the average means were evaluated using the Tukey multiple comparison test. Differences among means were considered significant when *p* < 0.05. Experimental data obtained from TPC, DPPH, FRAP, proximate composition, cooking loss, and textural properties were evaluated with one‐way analysis of variance (ANOVA). The results were expressed as mean values with standard errors (SE) of the mean.

## 3 Results and Discussion

### 3.1 Antioxidant Capacity Assay Results of Purslane, Arugula, Cress, Capers, and Linseed Extracts

The results of TPC, DPPH, and FRAP are presented in Table 1. The phenolic content found in purslane (P) and capers (CA) extracts was found to be higher than linseed (L), arugula (AR) and cress (CR) extracts (*p* < 0.05). There was no significant difference regarding the phenolic content of P and CA extracts. However, the lowest TPC value was obtained in the CR extract (*p* < 0.05). The highest FRAP values were obtained in the P extract, while the lowest FRAP value was obtained in the L extract (*p* < 0.05). No difference existed among CA, CR, and AR extracts in the case of FRAP values. No difference was also evident in DPPH values of P, CA, and AR extracts. The highest (*p* < 0.05) DPPH value was obtained in the L extract, followed by the CR extract, which had a higher DPPH value compared to the other extracts. DPPH values of P, CA, and AR were quite similar to each other. Based on TPC and FRAP results, it can be stated that P and CA extracts may possess stronger antioxidant power than L, AR, and CR extracts. Lim and Quah (2007) reported that TPC, DPPH scavenging (IC50) and FRAP values in cultivars of Purslane (*Portulaca oleracea L*.) varied from 127 to 478 mg GAE/100 g fresh weight of plant, 0.89 to 3.41 mg/mL, and 0.93 to 5.10 mg GAE/g, respectively. In another study, TPC, DPPH scavenging (IC50) and FRAP values in the same cultivar ranged from 174.5 to 348.5 mg GAE/100 g, 1.30 to 1.71 mg/mL, and 1.8 to 4.3 mg GAE/g, respectively (Uddin et al. 2012). When our results are compared with these previous studies, TPC values obtained in our study were higher, while FRAP and IC50 values were similar to those in previous studies. The differences in TPC results can be attributed to differences in sample pre‐treatment, solvent‐to‐sample ratio, solvent type used, extraction duration, and temperature conditions (Spigno et al. 2007).

**TABLE 1:** Evaluation of antioxidant activity in purslane, arugula, cress, capers, and linseed extracts.

Plant extracts	Total phenolic content (mg GAE/g)	FRAP (mM Fe^2+^/g)	IC_50_ mg/mL
P	18.54 ± 0.10^a^	255.70 ± 7.00^a^	0.02 ± 0.00^c^
CA	18.52 ± 0.12^a^	138.85 ± 5.00^b^	0.10 ± 0.01^c^
L	14.89 ± 0.19^b^	55.66 ± 4.00^c^	11.13 ± 1.00^a^
AR	12.86 ± 0.15^c^	123.69 ± 3.00^b^	0.08 ± 0.01^c^
CR	10.07 ± 0.20^d^	147.03 ± 3.00^b^	3.52 ± 0.10^b^

*Note:* Results are expressed as mean ± SE. ^a–d^Means within a column with unlike superscript letters are different statistically (*p* < 0.05).

Abbreviations: AR, arugula; CA, capers; CR, cress; L, linseed; P, purslane.

### 3.2 Effects of Purslane and Capers Extracts on Lipid Oxidation Inhibition and Physicochemical Features of Cooked Ground Beef During Refrigerated Storage

#### 3.2.1 Cooking Loss Results

The cooking loss values (Table 2) varied from 20.13% to 22.40%. The highest (*p* < 0.05) cooking loss was obtained in the treatments containing 2% P or CA extracts, whereas the 1% incorporation level of these two extracts resulted in similar cooking loss values determined in control. Our results were supported by Şimşek and Kilic (2024) who reported an increased cooking loss due to pomegranate peel extract addition in ground beef. Researchers emphasized that the incorporation of pomegranate peel extract caused a decrease in pH, which might be the reason for an increase in cooking loss. It is worth noting that initial meat pH strongly correlated with meat water holding capacity, drip loss, and cooking loss. This implies that a decrease in meat pH may cause a reduction in the water holding capacity of the meat, because of decreased protein solubility, consequently leading to increased cooking loss (Jankowiak et al. 2021). Although the cooking loss values were similar among control, P1, and CA1 treatments, control and P1 treatments demonstrated a higher pH value compared to CA1 (Table 3). Results revealed that the CA2 treatment displayed the lowest pH, consequently leading to the highest cooking loss values among all treatments (*p* < 0.05). The relationship between pH and cooking loss is influenced by factors such as the intrinsic factors in the muscle structure and the types and concentrations of the plant extracts used. Reduced pH values generally impact protein solubility, which could potentially decrease water holding capacity and increase cooking loss (Kılıç et al. 2014). Even though increasing P and CA extract concentration from 1% to 2% caused a slight increase in cooking loss values, this increasing cooking loss can be regarded as a negligible factor for limiting the overall acceptability of the final product. When considering the progress made in inhibiting lipid oxidation.

**TABLE 2:** Proximate composition and cooking loss values of cooked ground beef.

Treatments	Moisture (%)	Protein (%)	Fat (%)	Ash (%)	Cooking loss (%)
Control	68.70 ± 0.27^a^	22.41 ± 0.22^bc^	5.25 ± 0.35^ab^	3.52 ± 0.06^a^	20.33 ± 0.40^c^
P1	68.71 ± 0.63^a^	22.20 ± 0.46^c^	5.50 ± 0.35^a^	3.44 ± 0.13^a^	20.98 ± 0.18^bc^
P2	68.41 ± 0.32^a^	23.13 ± 0.17^ab^	4.98 ± 0.22^abc^	3.38 ± 0.08^a^	21.64 ± 0.35^ab^
CA1	68.78 ± 0.19^a^	23.02 ± 0.15^abc^	4.44 ± 0.17^bc^	3.64 ± 0.03^a^	20.13 ± 0.19^c^
CA2	68.70 ± 0.30^a^	23.41 ± 0.21^a^	4.37 ± 0.21^c^	3.44 ± 0.11^a^	22.40 ± 0.18^a^

*Note:* Results are expressed as mean ± SE. ^a–c^Means within a column with unlike superscript letters are different statistically (*p* < 0.05). Control: cooked ground beef without extract, P1: cooked ground beef containing 1% purslane extract, P2: cooked ground beef containing 2% purslane extract, CA1: cooked ground beef containing 1% capers extract and CA2: cooked ground beef containing 2% capers extract.

**TABLE 3:** Effects of purslane and capers extracts on CIE L*, a*, b* color and pH values of cooked ground beef during refrigerated storage.

Treatments	L*			a*			b*			pH		
	Processing day	Day 5	Day 10	Processing day	Day 5	Day 10	Processing day	Day 5	Day 10	Processing day	Day 5	Day 10
Control	51.98 ± 0.26^b^	52.36 ± 0.21^ab^	52.93 ± 0.19^ab^	13.71 ± 0.22^ab^	13.36 ± 0.52^b^	12.57 ± 1.05^bc^	2.94 ± 0.20^fg^	2.62 ± 0.21^fg^	3.53 ± 0.57^defg^	5.75 ± 0.01^ef^	5.90 ± 0.01^a^	5.90 ± 0.01^a^
P1	52.34 ± 0.17^ab^	52.57 ± 0.35^ab^	53.59 ± 0.62^a^	13.60 ± 0.14^ab^	15.54 ± 0.38^a^	10.64 ± 0.36^cde^	3.43 ± 0.24^defg^	2.47 ± 0.23^g^	3.72 ± 0.17^def^	5.77 ± 0.00^e^	5.88 ± 0.01^ab^	5.88 ± 0.01^b^
P2	51.77 ± 0.32^b^	51.83 ± 0.20^b^	53.14 ± 0.26^ab^	12.77 ± 0.21^b^	12.43 ± 0.35^bcd^	10.74 ± 0.10^cde^	3.25 ± 0.19^efg^	3.37 ± 0.16^efg^	4.20 ± 0.18^cde^	5.73 ± 0.00^fg^	5.82 ± 0.01^c^	5.84 ± 0.00^c^
CA1	52.31 ± 0.27^ab^	52.22 ± 0.33^ab^	52.90 ± 0.31^ab^	11.93 ± 0.35^bcd^	12.83 ± 0.35^b^	10.48 ± 0.36^de^	3.46 ± 0.18^def^	3.77 ± 0.18^def^	4.57 ± 0.34^bcd^	5.69 ± 0.00^h^	5.79 ± 0.00^d^	5.75 ± 0.00^ef^
CA2	52.09 ± 0.26^b^	51.97 ± 0.23^b^	52.81 ± 0.16^ab^	10.78 ± 0.23^cde^	9.83 ± 0.14^e^	9.67 ± 0.12^e^	5.14 ± 0.17^abc^	5.61 ± 0.19^ab^	5.81 ± 0.12^a^	5.62 ± 0.01^i^	5.71 ± 0.00^gh^	5.70 ± 0.00^h^

*Note:* Results are expressed as mean ± SE. ^a–i^Means with unlike superscript letters are different statistically (*p* < 0.05) for each of tested parameters specified in the table. Control: cooked ground beef without extract, P1: cooked ground beef containing 1% purslane extract, P2: cooked ground beef containing 2% purslane extract, CA1: cooked ground beef containing 1% capers extract and CA2: cooked ground beef containing 2% capers extract.

#### 3.2.2 Physicochemical Properties of Cooked Ground Beef

The proximate composition (moisture, fat, protein and ash) values of the cooked ground beef are presented in Table 2. It was observed that the incorporation of both P and CA extracts had no impact on the moisture and ash values when compared to control. But the protein contents of ground beef samples containing 1% CA or 1%–2% P extracts closely resembled that of control. However, in the case of ground beef containing 2% CA extract, the protein content was higher when compared with control (*p* < 0.05). In a previous study using powders obtained from different parts of the capers plant, it was determined that the capers bud had more protein content than other parts of the plant (Abu‐shama 2022). Ground beef samples containing 1%–2% P or 1% CA extracts exhibited similar fat levels to those of control, whereas the sample containing 2% CA extract had a lower fat level compared to control (*p* < 0.05). It has been stated that the addition of capers may cause a decrease in fat content as a result of its increasing effect on dry matter content (Yerlikaya and Karagozlu 2014).

pH results presented in Table 3 indicated that, in general, there was an increase (*p* < 0.05) in pH values of cooked ground beef across all treatments throughout the refrigerated storage period. Ibrahim et al. (2010) also found that lamb patties showed an increasing pH during refrigeration, linking this rise to ammonia buildup due to protein denaturation. Similar pH increases during the storage of pork meatballs and chicken meatballs incorporated with pomegranate peel extract were also reported previously (Lee and Chin 2012; Sharma and Yadav 2020). Regardless of plant extract addition ratio, the incorporation of both P and CA extracts caused pH decline when compared to control on processing day. This trend was also evident throughout day 5 and day 10 (*p* < 0.05), except for the CA1 treatment which had similar pH with control on day 5. Furthermore, the incorporation of CA extract caused more pH decline compared to P addition regardless of the incorporation dose (*p* < 0.05). Moreover, an increasing incorporation ratio of these two extracts resulted in lower pH in ground beef during both the processing day and refrigerated storage periods (*p* < 0.05). Previous study revealed the presence of steroids, alkaloids, tannins, carbohydrates, saponins, flavonoids, and phenols in the aqueous CA extract (Shahrajabian et al. 2021). Additionally, purslane is recognized as one of the richest plants as far as the content of fatty acids, especially linoleic and α‐linolenic acids (Montoya‐García et al. 2023). Thus, the observed pH reduction in ground beef samples upon the addition of P and CA extracts may be associated with the existence of acidic components, particularly tannins and phenols, in the plant extracts (Mustafa 2012; Saffaryazdi et al. 2020; Shahrajabian et al. 2021). Furthermore, as proteins in cooked ground beef gradually degrade during storage, they release amino acids into the surrounding environment, and this process may contribute directly to the observed pH increase over time (Köker et al. 2024).

The findings regarding the color attributes of the ground beef treatments are provided in Table 3. No differences were obtained in L* values among all treatments on the processing day and throughout the storage durations. Moreover, all treatments exhibited quite stable L* values during storage, indicating consistent lightness over time. As far as a* values are concerned, on the processing day, CA2 treatment had lower (*p* < 0.05) a* values compared to control, P1, and P2 treatments, which were similar to each other. The relationship between meat pH and color may be an important factor affecting a* values determined in the present study. A decrease in meat pH is known to be associated with a reduction in a* values, as lower pH can lead to increased metmyoglobin levels and decreased myoglobin, which reduces redness (Jankowiak et al. 2021). This negative correlation between pH and metmyoglobin levels suggests that lower pH values accelerate the oxidation of myoglobin to metmyoglobin, as oxidative processes like myoglobin and hemoglobin oxidation occur more rapidly in acidic conditions (Hoa et al. 2021; Yin et al. 2017; Zareian et al. 2019). Moreover, a general decrease (*p* < 0.05) in a* values was evident in treatments with 1%–2% P extract throughout the storage, whereas other treatments were stable during the same period. At the end of storage, CA1 and CA2 treatments had lower (*p* < 0.05) a* values than control, indicating a reduction in redness. However, the incorporation of P extract did not influence a* values compared to control. Regarding b* values, CA2 treatment exhibited higher b* values on the processing day and throughout the storage period compared to other treatments, which were quite similar to each other. The higher b* values in the CA2 treatment are thought to be due to the dark brown color of CA extract, which likely influenced the yellowness of the meat.

The texture profile analysis results (Table 4) revealed that the extracts used as additives did not significantly impact the hardness, adhesiveness, resilience, and springiness values of the ground beef. However, it was determined that cooked ground beef with 2% CA extract showed lower chewiness compared to the control (*p* < 0.05). This indicates that cooked ground beef with 2% CA extract might have a softer and less chewy texture compared to the control. Furthermore, the treatments with 1%–2% CA extract had lower (*p* < 0.05) cohesiveness compared to those with 2% P extract. This suggests that ground beef with CA extract might be less cohesive, or less likely to hold together, compared to those with P extract at the same concentration. Overall, these results demonstrated that CA extract seems to have a notable effect on chewiness and cohesiveness, while the other texture attributes remained unaffected. These textural changes may be explained by the effect of plant‐based extracts on the protein oxidation in muscle foods. Ganhão et al. (2010) reported that phenolics derived from the plant extracts would be able to reduce some textural parameters of hamburger patties via inhibition of protein oxidation.

**TABLE 4:** Effects of purslane and capers extracts on textural properties of cooked ground beef.

Treatments	Hardness (*N*)	Adhesiveness (mJ)	Resilience	Cohesiveness	Springiness	Chewiness (*N*)
Control	7.54 ± 0.24^a^	6.37 ± 0.52^a^	0.05 ± 0.00^a^	0.42 ± 0.01^ab^	0.95 ± 0.01^a^	2.99 ± 0.14^a^
P1	6.77 ± 0.53^a^	5.78 ± 0.54^a^	0.05 ± 0.01^a^	0.41 ± 0.02^ab^	0.93 ± 0.01^a^	2.62 ± 0.27^ab^
P2	6.73 ± 0.37^a^	6.03 ± 0.70^a^	0.05 ± 0.00^a^	0.44 ± 0.01^a^	0.94 ± 0.01^a^	2.77 ± 0.12^ab^
CA1	6.99 ± 0.49^a^	6.25 ± 0.61^a^	0.05 ± 0.00^a^	0.39 ± 0.01^b^	0.94 ± 0.01^a^	2.55 ± 0.22^ab^
CA2	6.32 ± 0.32^a^	5.73 ± 0.28^a^	0.04 ± 0.00^a^	0.38 ± 0.02^b^	0.94 ± 0.01^a^	2.25 ± 0.20^b^

*Note:* Results are expressed as mean ± SE. ^a,b^Means within a column with unlike superscript letters are different statistically (*p* < 0.05). Control: cooked ground beef without extract, P1: cooked ground beef containing 1% purslane extract, P2: cooked ground beef containing 2% purslane extract, CA1: cooked ground beef containing 1% capers extract, and CA2: cooked ground beef containing 2% capers extract.

#### 3.2.3 TBARS and LPO

Table 5 presents TBARS and LPO results of the cooked ground beef, as well as their changes over 10‐day storage period at +4°C. TBARS and LPO values in all treatments increased (*p* < 0.05) over the course of 10‐day storage. Nevertheless, it was observed that TBARS formation was significantly inhibited in treatments with P or CA extracts when compared to the control over the storage (*p* < 0.05). Increasing the incorporation ratio of P or CA extracts decreased TBARS in cooked ground beef during the storage. The highest TBARS values existed in the control, meantime the lowest TBARS were obtained in the CA2 treatment (*p* < 0.05). Reduction rates in TBARS formation in P1, P2, CA1, and CA2 treatments were 33.71%, 37.37%, 28.64%, and 55.70%, respectively, when compared with the control. Except for the processing day, the highest and the lowest LPO values were detected in the control and CA2 treatments throughout the storage, respectively (*p* < 0.05). Results also revealed that the CA extract was more effective than the P extract in reducing LPO formation during storage (*p* < 0.05). In addition, the P1, P2, CA1, and CA2 treatments reduced lipid oxidation by 32.59%, 51.11%, 43.04%, and 58.42%, respectively, compared to the control. The plant extracts were reported to have an influence on enhancing the oxidative stability in muscle foods (Hanczakowska et al. 2015). Tocopherols, gallic acid, flavonoids, sesamol, ferulic acid, and epicatechin are among the prevalent phenolic antioxidants derived from plant sources. The extract of caper demonstrated that the aqueous extract was found to contain flavonoids, phenols, steroids, carbohydrates, saponins, alkaloids, and tannins (Mustafa 2012; Shahrajabian et al. 2021). Similarly, purslane contains terpenoids, alkaloids, glutathione, flavonoids, and volatile oils (Saffaryazdi et al. 2020).

**TABLE 5:** Effects of purslane and capers extracts on TBARS and LPO values in cooked ground beef during refrigerated storage.

Treatments	Processing day	Day 5	Day 10
TBARS (μmol MDA/kg)			
Control	8.36 ± 0.20^ij^	21.52 ± 0.42^b^	26.01 ± 0.72^a^
P1	6.30 ± 0.12^jk^	14.29 ± 0.46^ef^	17.24 ± 0.30^cd^
P2	5.53 ± 0.19^k^	13.34 ± 0.52^fg^	16.29 ± 0.39^de^
CA1	5.35 ± 0.16^k^	14.24 ± 0.64^ef^	18.56 ± 0.76^c^
CA2	4.63 ± 0.12^k^	9.43 ± 0.14^hi^	11.52 ± 0.26^gh^
LPO (μmol/kg LPO)			
Control	54.10 ± 2.05^i^	175.57 ± 3.18^b^	236.81 ± 2.68^a^
P1	44.51 ± 1.22^ij^	116.49 ± 2.50^e^	159.61 ± 4.00^c^
P2	32.41 ± 0.87^k^	88.62 ± 1.54^g^	115.76 ± 3.88^e^
CA1	47.84 ± 0.75^i^	109.74 ± 1.19^ef^	134.88 ± 2.45^d^
CA2	34.11 ± 1.22^jk^	72.07 ± 1.24^h^	98.45 ± 2.04^fg^

*Note:* Results are expressed as mean ± SE. ^a–k^Means with unlike superscript letters are different statistically (*p* < 0.05) for each of tested parameters specified in the table. Control: cooked ground beef without extract, P1: cooked ground beef containing 1% purslane extract, P2: cooked ground beef containing 2% purslane extract, CA1: cooked ground beef containing 1% capers extract and CA2: cooked ground beef containing 2% capers extract.

Additionally, other plant compounds like essential oils, tannic acid, and carotenoids are recognized for their potent antioxidant properties (Huang and Ahn 2019). Wang et al. (2021) reported that meat samples treated with 0.1%, 0.3%, and 0.5% P extract showed low lipid and protein oxidation. In a study involving various plant (Dangshen, platycodon, capillary wormwood, Chinese cinnamon, Chinese rhubarb, jujube, cape jasmine, true sandalwood, female ginseng, and Chinese ground orchid) extracts, it was observed that TBARS values of all the plant extracts used remained lower than those of the control throughout 11‐day storage (4°C) period (Luo et al. 2007). Another study reported that sausage samples with rosemary extract displayed reduced TBARS values when compared to the control following 14‐day storage (4°C) period (Sebranek et al. 2005). In a study with a similar approach, it was established that goat meat treated with peony (white, moutan and red), angelica, rehmania, and sappanwood extracts exhibited a strong antioxidant effect on cooked–stored goat meat (Han and Rhee 2005).

#### 3.2.4 Microbiological Analysis Results

Cooking is considered the most effective method for destroying microorganisms that cause diseases in meat and meat products (Jezek et al. 2019). In all treatment groups, the total mesophilic aerobic bacteria, total coliform bacteria, and yeast‐mold counts were below the detection limit. No microbial growth was also detected during the storage period in all treatments. Heat treatment of meat products should ensure the elimination or significant reduction of vegetative microbiota. Pasteurization, typically between 72°C and 80°C, is effective in destroying vegetative forms of microorganisms. This high temperature drastically alters the microbiota composition, killing non‐spore pathogenic and most saprophytic microorganisms (cocci, lactic acid bacteria, yeast), opportunistic microorganisms, vegetative forms, and some spores of spore‐forming bacteria (Vinnikova and Kyshenia 2019). The inhibitory effect of exposed heat treatment is thought to be the reason for the no‐growth of the total mesophilic aerobic bacteria, total coliform bacteria, and yeast‐mold.

## 4 Conclusion

The results of in vitro antioxidant potential of P, AR, CR, CA, and L extracts revealed that P and CA extracts exhibited higher antioxidant activity compared to other extracts, particularly in terms of TPC, FRAP, and DPPH results. Furthermore, evaluation of P and CA extracts as a natural antioxidants in cooked ground beef indicated that both P and CA extracts reduced pH levels and effectively inhibited lipid oxidation during 10 days of refrigerated storage, with CA extract showing particularly stronger antioxidative effects. The antioxidant activity provided by both extracts exhibited a dose‐dependent trend, with higher incorporation levels (2%) leading to greater lipid oxidation inhibition. Even though incorporation of both extracts at 2% increased cooking loss, a 1% incorporation rate did not have any influence on cooking loss. Furthermore, neither extract influenced the color or texture of ground beef at a 1% incorporation rate. However, a 2% CA extract addition slightly altered certain quality parameters, such as reducing redness and chewiness while increasing yellowness. Overall, these findings highlight the potential of P and especially CA extracts as natural antioxidants in meat products, offering an effective means of enhancing oxidative stability without compromising main physicochemical properties when used at optimal concentrations.

## Author Contributions

**Hanife Bayındır:** conceptualization (equal), data curation (equal), formal analysis (equal), investigation (equal). **Birol Kılıç:** conceptualization (equal), data curation (equal), funding acquisition (equal), methodology (equal), project administration (lead), supervision (lead), validation (equal), writing – original draft (equal), writing – review and editing (equal). **Melis Yıldız:** conceptualization (equal), investigation (equal), methodology (equal), software (equal), writing – original draft (equal), writing – review and editing (equal).

## Conflicts of Interest

The authors declare no conflicts of interest.

## Data Availability

Data will be made available on request.
